# Digit loss due to *Demodex* spp. infestation in a dog: clinical and pathological features

**Published:** 2013-05-20

**Authors:** F. Grandi, A. Pasternak, H.E.O. Beserra

**Affiliations:** 1 *Department of Diagnostic Pathology, Public Veterinary Hospital, Veterinary Service of National Association of Small Animal Clinicians (ANCLIVEPA), São Paulo, SP, Brazil*; 2 *Department of Veterinary Clinics, School of Veterinary Medicine and Animal Science, Univ. Estadual Paulista – UNESP, Botucatu, SP, Brazil*; 3 *Department of Pathology, Botucatu Medical School, Univ. Estadual Paulista – UNESP. Botucatu, São Paulo, Brazil*; 4 *Department of Dermatology, Public Veterinary Hospital, Veterinary Service of National Association of Small Animal Clinicians (ANCLIVEPA), São Paulo, SP, Brazil*

**Keywords:** Acaricidal treatment, Canine, Parasite, Pododemodicosis, Skin

## Abstract

Here we describe a rare clinical manifestation of canine pododemodicosis. A dog was presented with pedal erythema, scaling, crusting, severe edema and digit loss. The following diseases were taken into account for the differential diagnosis: pododemodicosis, lethal acrodermatitis, zinc responsive dermatosis and pemphigus foliaceus. Results from skin biopsies revealed the presence of *Demodex* spp. of mites in the follicular infundibula and a severe inflammatory process (pododemodicosis). Upon the acaricidal treatment, the patient exhibited favorable signs of clinical improvement.

## Introduction

Canine demodicosis is a non-contagious parasitic skin disease caused by an overpopulation of the host-specific follicular mite, *Demodex* spp. Clinically, demodicosis is often divided into two broad categories based on the extent of the area of the skin that is affected due to the infestation: a) generalized demodicosis, a potentially life-threatening disease, and b) localized demodicosis (Gross *et al.*, 2005; Mueller *et al.*, 2012).

Pododemodicosis is a clinical manifestation that is characterized by severe involvement of the feet and occurs either as an isolated syndrome or is secondary to a generalized infection. Dermatological lesions include erythema, swelling, secondary deep pyoderma, scarring of digits, interdigital webs, ventral pawpads, and around the clawbeds (Gross *et al.*, 2005).

Diagnosis is often straightforward and poses no challenges to clinicians, because skin scrapings and biopsies readily demonstrate mites as the causative agents. In this report, we describe a previously unreported case of a digit loss associated with pedal demodicosis in a dog (Ravera *et al.*, 2013).

## Case details

**Fig. 1:**
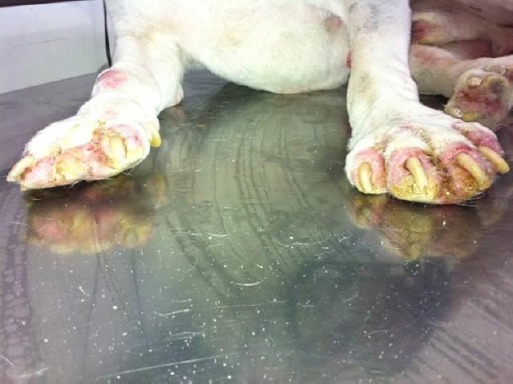
A gross photograph of the examined dog, Bull Terrier, prior to the treatment. The “flat feet” appearance and the loss of the digit in the right thoracic limb are clearly evident.

A 4-year-old male, white-coated Bull Terrier dog suffering from a chronic pododermatitis, difficulty in walking and with reduced appetite was presented to the Public Veterinary Hospital. The case history revealed that the onset of clinical signs dates back to a year prior to the presentation of the case. Clinical observations revealed that all the feet were swollen, erythematous, crusted, hyperkeratotic, ulcerated and painful with loss of a digit from the right thoracic limb (Fig. 1).

Examination also revealed that the footpads exhibited severe hyperkeratosis. Furthermore, the base of the tail was ulcerated and necrotic. Preputeal skin, lips and periocular region had papules, pustules and crusts with secondary *Malassezia* spp. infection which was detected by cytological examination. The differential diagnosis included diseases such as pododemodicosis, zinc responsive dermatosis, lethal acrodermatitis of the Bull Terriers (LABT) and pemphigus foliaceus.

The results from skin scrapings were inconclusive. Thus, the animal was initially treated with penthabiotic (Shotapen^®^ L.A. – Virbac^®^ (procaine benzylpenicillin 10,000,000 IU + benzathine benzylpenicillin 10.000,000 IU + dihydrostreptomycin 20 g; 0.1 mL/kg/every 4 days in three applications), dexamethasone (Cort-Trat^®^ SM – Santa Marina - 20mg/mL) (100 mg in a single dose), itraconazole (10mg/kg/day/PO) and supplemented with zinc (10mg/kg/day/PO) for 30 days. The animal was also subjected to periodic baths with 4.5% benzoyl peroxide once a week throughout the entire course of treatment.

However, even after thirty days past the initial diagnosis and treatment, there were no clinical signs of improvement. Thus, a skin biopsy was performed for a further detailed diagnosis and to identify the causative agent. Skin biopsy from the affected digits revealed mild parakeratosis, acanthosis, spongiosis, intraepidermal micropustules that are composed of neutrophils and mild epidermal pallor. Superficial and deep dermis presented a diffuse inflammatory infiltrate composed of lymphocytes, plasma cells, histiocytes, neutrophils and rare eosinophils and furunculosis.

**Fig. 2:**
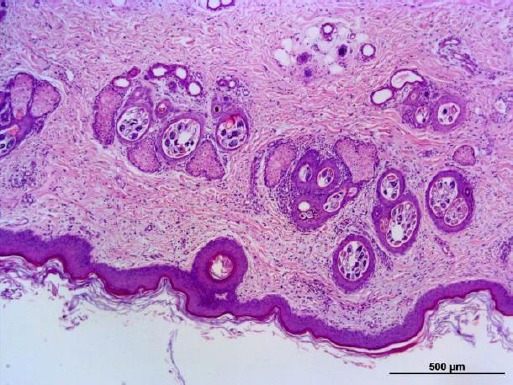
Histological section from the skin biopsy of the case under investigation. Various stages of the *Demodex* spp. of mites can be clearly visualized in the hair follicles (H&E staining, 50x magnification).

Various parasitic stages of *Demodex* spp. mites were found in the hair follicles (Fig. 2), presenting us a clear evidence of pododemodicosis. Following this definitive diagnosis, dermatologist recommended an acaricidal treatment with ivermectin (0.5 mg/kg/daily) for 90 days and daily medicinal baths with amitraz (Delayte *et al.*, 2006; Mueller *et al.*, 2012).

A follow up of the case thirty days later revealed that there was a mild overall improvement in the clinical condition as characterized by reduced erythema and edema. These clinical signs allowed us to perform a new skin biopsy which further revealed compact orthokeratotic hyperkeratosis and acanthosis; hyperplastic and hyperkeratotic follicular infundibula; diffuse piogranulomatous inflammatory infiltrate composed of neutrophils, plasma cells and histiocytes in the superficial and deep dermal layers. Mites were extracted from the hair follicles. Results from Periodic acid-Schiff and Ziehl-Neelsen staining’s were inconclusive.

**Fig. 3:**
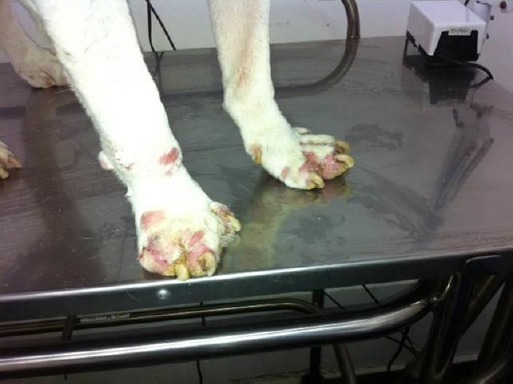
A gross photograph of the examined dog after the acaricidal treatment. Note the significant improvement in the appearance of the feet after 4 weeks of the treatment. Also, loss of the digit in the right thoracic limb is clearly evident in this photograph.

A diagnosis of pododemodicosis was made based on the various evidences stated above. In this context, the owner of the dog was not advised to start a different treatment regimen. Instead, the dermatologist recommended the repetition of the acaricidal treatment for an additional 90 days. Interestingly, just 4 weeks into the repetition of the treatment, the animal exhibited nearly 85% clinical improvement (Fig. 3). The patient still remained under veterinary care and supervision for a complete cure.

## Discussion

To our knowledge, we present the first explicit evidence to show that loss of digits can occur as a result of pododemodicosis in canines. Clinical manifestations are the key indicators for proper dermatological evaluation of the cases with skin lesions. In the present case, severe damages to the digits and rapid clinical aggravation of the symptoms were detected. A significant finding from our current case is the clinical picture pertaining to the digit loss, a finding that has not been previously described/reported in the literature (Gross *et al.*, 2005; Gortel, 2006; Mueller, 2012).

Results from skin scrapings were inconclusive in this case. One possible reason for such inconclusive results is that it might be difficult to diagnose the disease just by using skin scrapings in dogs with severe pododemodicosis. This is because the extensive scarring as observed in this case or in cases such as foreign body granulomas in furunculosis preclude the use of skin scrapings to conclusively establish the causative factors of the disease (Gross *et al.*, 2005; Gortel, 2006). Histopathological findings, coupled with the lack of clinical improvement to dietary supplementation with zinc enabled us to exclude LABT and zinc responsive dermatosis, because both the types of diseases can produce strikingly similar symptoms (Gross *et al.*, 2005). In the present study, we could not precisely determine the pathogenesis of the digit loss. A presumptive conclusion on this condition is more or less an outcome of secondary vascular lesions such as thrombosis and/or direct vascular damage induced by severe inflammatory reactions associated with folliculitis and furunculosis or even trauma. We recommend that preparing serial histological sections of the affected digits at different time points during the disease progression might aid in determining the exact mechanism that underlies the digit loss in cases of pododemodiciosis.
